# Anthrax in Eastern Turkey, 1992–2004

**DOI:** 10.3201/eid1112.050779

**Published:** 2005-12

**Authors:** Zülal Özkurt, Mehmet Parlak, Rustu Tastan, Ufuk Dinler, Yavuz S. Saglam, Serhat F. Ozyurek

**Affiliations:** * Ataturk University, Erzurum, Turkey; † University of Kocaeli, Kocaeli, Turkey; ‡ Institute of Veterinary Control and Research, Erzurum, Turkey; § Health Directorate, Erzurum, Turkey

**Keywords:** anthrax, Bacillus anthracis, epidemiology, epizootiology, human, Turkey, dispatch

## Abstract

We investigated animal and human anthrax cases during a 13-year period in eastern Turkey. From 1992 to 2004, a total of 464 animal and 503 human anthrax cases were detected. Most cases occurred in summer. Anthrax remains a health problem in eastern Turkey, and preventive measures should be taken.

Anthrax is an endemic zoonosis in Turkey, but the incidence of the disease has been decreasing. From 1960 to 1969, a total of 10,724 human cases were reported compared to 4,423 cases from 1980 to 1989. After 1990, the number of human anthrax cases was <300 annually (*1*). Animal anthrax cases have also been decreasing, and 277 cases were reported in 2001; 218 in 2002 and 72 in the first 8 months of 2003. We conducted this study to investigate the epizootiology and of epidemiology of anthrax during the 13-year period from 1992 through 2004 in eastern Turkey.

## The Study

Animal anthrax cases from the Institute of Veterinary Control and Research in Eastern Anatolia Region and human cases from the Department of Clinical Bacteriology and Infectious Diseases (in the tertiary hospital) and state health centers or hospitals (primary and secondary health care centers) from January 1992 to November 2004 were included. Data were collected from formal records.

A suspected case of cutaneous anthrax is characterized by a skin lesion evolving from a papule, through a vesicular stage, to a depressed black eschar; edema, erythema, or necrosis without ulceration may be present. A confirmed case is defined through positive smear or isolation of *Bacillus anthracis* in clinical specimens (*2*). *B. anthracis* isolates were identified on the basis of conventional methods such as gram-positive bacilli with spores seen in smear, the presence of a capsule, lack of motility, and catalase positivity.

In humans, the diagnosis of anthrax was based on clinical findings or microbiologic procedures, including Gram stain (short chains of capsulated gram-positive bacilli seen on a smear) and isolation of *B. anthracis* from a clinical specimen (*3*). In animals, the diagnosis was made by examining the history, autopsy findings, and Gram stain or cultures from tissues (liver, spleen, lymph node, bone marrow, and ear) of a sick animal.

**Figure 1:**
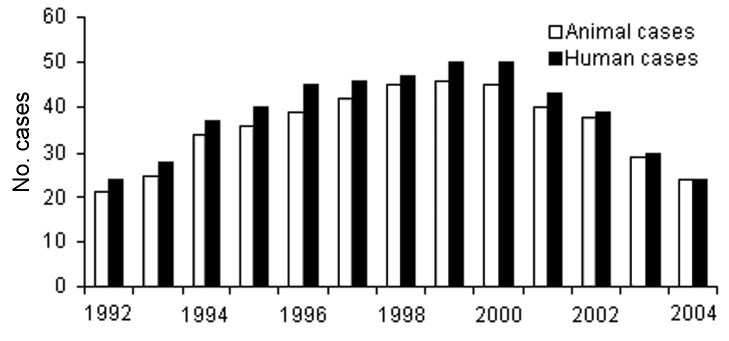
Annual distribution of anthrax cases.

From the 13-year period January 1992–November 2004, a total of 464 animal and 503 human cases of anthrax were detected in eastern Turkey. Of 464 animal cases, 20 (4.3%) were sheep, and 444 (95.7%) were cattle. The mean number of cases was 35.6 per year in animals and 38.6 per year in humans. Anthrax cases in both humans and animals increased from 1993 to 1999 and decreased after 2000 (Figure 1).

Most animal (319 [68.7%]) and human (338 [67.2%]) cases occurred between July and October. Anthrax was seen most frequently in Erzurum and Kars, cities that are centers of animal commerce (Table).

**Table:** Distribution of anthrax cases in cities, eastern Turkey

City	No. human cases	No. animal cases
Agri	35	28
Ardahan	45	39
Artvin	13	15
Bayburt	30	47
Erzincan	24	23
Erzurum	198	170
Igdir	31	29
Gumushane	24	19
Kars	103	94
Total	503	464

**Figure 2:**
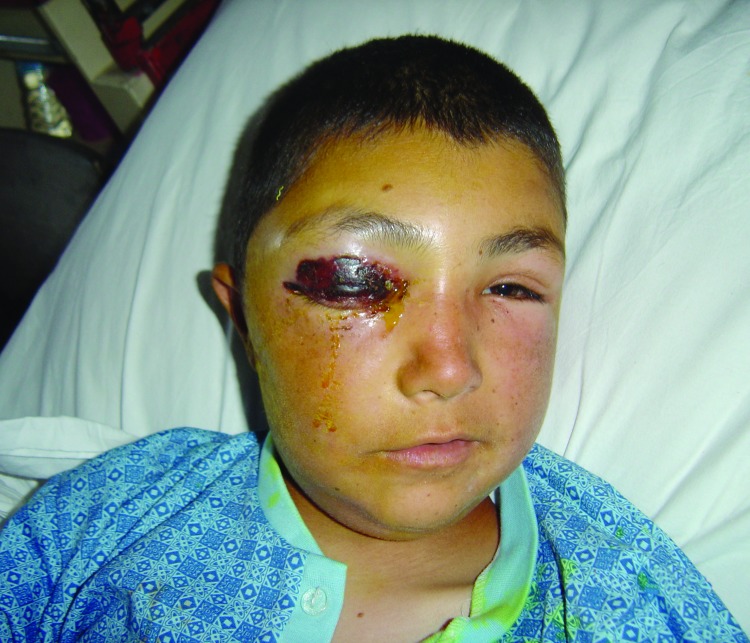
Cutaneous anthrax on eyelids. Photographer: Zülal Özkurt. Photograph taken with patient's permission.

All animal cases died. Most of the human cases were cutaneous anthrax (Figure 2) Only 2 cases (0.39%) died, one from meningitis, and the other from asphyxia due to extensive anthrax edema (*4**,**5*). The remaining patients recovered. All the patients had a history of exposure to anthrax-infected animals.

## Conclusions

Anthrax is endemic in the Middle East, some Asian countries, Africa, and South America. The disease has also been detected in Turkey (*6**–**8*). In eastern Turkey, most people live in rural areas and work in agriculture and stockbreeding. Animals usually graze in pasture from April through November. In this study, most anthrax cases were seen from April to November. Similar seasonal distribution has been observed in other studies (*5**,**9**–**11*).

The numbers of both animal and human anthrax cases in eastern Turkey increased from 1995 to 2000. Nevertheless, from 2000 until 2004, cases have been decreasing. Economic and social changes, strict animal vaccination programs, and education of farmers may have contributed to this trend. Anthrax was most commonly seen in Erzurum and Kars, which are centers of animal trade and have large international commercial roads.

Skinning, butchering a sick animal, and handling and eating contaminated meat are known risk factors for human anthrax (*12*). All patients in our study had a history of exposure to anthrax-infected animals. Although some patients had eaten infected meat, no gastrointestinal anthrax cases occurred, which may be due to the cooking methods these patients used (overcooking the meat). However, humans should not eat meat from a sick animal.

In this study, more anthrax cases occurred in humans than in animals. Several factors could account for this finding. First, sometimes sick animals have been butchered by humans and are not reported to veterinary institutions, so some animal cases are not recorded. Secondly, 1 sick animal can contaminate several persons who participate in the slaughtering procedure. Finally, because fewer resources are available for the veterinary infrastructure and reporting mechanisms than for the public health system, animal cases are probably underreported. Similar results have been reported in other studies (*7**,**8*). For example, Aydin et al. (*8*) detected 164 animal anthrax cases versus 327 human cases in 1993, and 50 animal cases versus 445 human cases in 1994. Keçeci et al. (*7*) reported 17 animal versus 166 human anthrax cases in 1995. Otlu et al. (*13*) reported 45 animal cases versus 89 human anthrax cases in 2000–2001.

In this study, most animal anthrax cases occurred in cattle. Several factors may account for this occurrence. First, more cattle than sheep are found in the region. Second, cattle graze in plains, but sheep graze in high plateaus and slopes, so cattle probably have more exposure to environmental anthrax risks than sheep (spores accumulate more in plains). Third, cattle have more economic value than sheep; as a result, sick cattle are reported to the veterinary service and recorded. But, when a sheep becomes ill, it is slaughtered before dying or buried immediately after death; its death is not reported to the veterinary service in rural areas. Aydin et al. (*8*) reported that 72.9% of anthrax cases occurred in cattle and 27.0% in sheep in the same region in 1994. Otlu et al. (*13*) detected 11 anthrax cases in sheep versus 34 anthrax cases in cattle in the same region in 2000.

Good surveillance, decontamination and disinfection procedures, and education are mandatory to reduce the incidence of anthrax. Employees should be educated about the disease to reduce the risk for disease. Controlling the disease in humans ultimately depends on controlling it in animals by effective surveillance and immunization. The carcasses of all animals that have died with a confirmed diagnosis of anthrax should be thoroughly cremated, and the remains should be deeply buried (*14**,**15*).
